# Acquisition and visualization of 5D respiratory-resolved cardiac MRI

**DOI:** 10.1186/1532-429X-14-S1-P237

**Published:** 2012-02-01

**Authors:** Holden H Wu, Dwight G Nishimura, Bob S Hu, Michael V McConnell

**Affiliations:** 1 Cardiovascular Medicine, Stanford University, Stanford, CA, USA; 2 Electrical Engineering, Stanford University, Stanford, CA, USA; 3 Palo Alto Medical Foundation, Palo Alto, CA, USA

## Summary

We image the heart during free breathing and explicitly resolve 3D volumetric information over both the cardiac and respiratory cycles to capture a 5D view of the heart. This 5D dataset can be displayed in a variety of ways to study disease states that exhibit variations in cardiac function with respect to respiration, such as pericardial tamponade/constriction and diastolic dysfunction.

## Background

In conventional cardiac MRI, respiratory effects are suspended or counteracted to avoid artifacts. However, in many disease states, such as pericardial tamponade/constriction and diastolic dysfunction, it is precisely variations in cardiac morphology/function with respect to respiratory changes that reflect the pathophysiology and should be studied. In this work, we image the heart during free breathing and explicitly resolve a 3D volume over both cardiac and respiratory cycles. We explore a range of options for visualizing the information captured in this 5D state of the heart.

## Methods

**Figure 1:**
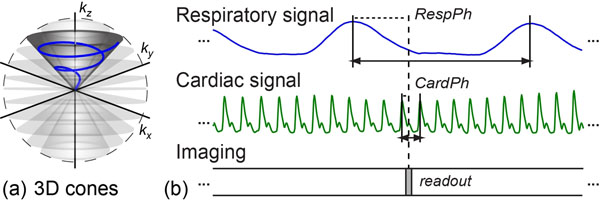
(a) 3D cones sampling trajectory. (b) The respiratory phase (*RespPh*) and cardiac phase (*CardPh*) of each readout are retrospectively determined from the recorded physiologic signals. Acquired data are then re-ordered and reconstructed to obtain a 5D dataset.

Free-breathing 5D cardiac MRI scans were conducted on a GE Signa 1.5 T Excite system. A non-Cartesian 3D cones sampling trajectory (Fig. 1a) was used to achieve 4-fold acceleration (vs. Cartesian) and provide greater robustness to motion/flow and undersampling. Data were obtained in the short-axis orientation with FOV of 36x36x8 cm^3^ and resolution of 2.4x2.4x8 mm^3^. Cardiac and respiratory signals were recorded concurrently during the scan (Fig. 1b) and used to retrospectively reorder the readouts into a dataset of dimensions [3D spatial information, 1D cardiac cycle, 1D respiratory cycle]. Total scan time ranged from 2 to 5 min depending on the respiratory rate.

## Results

**Figure 2:**
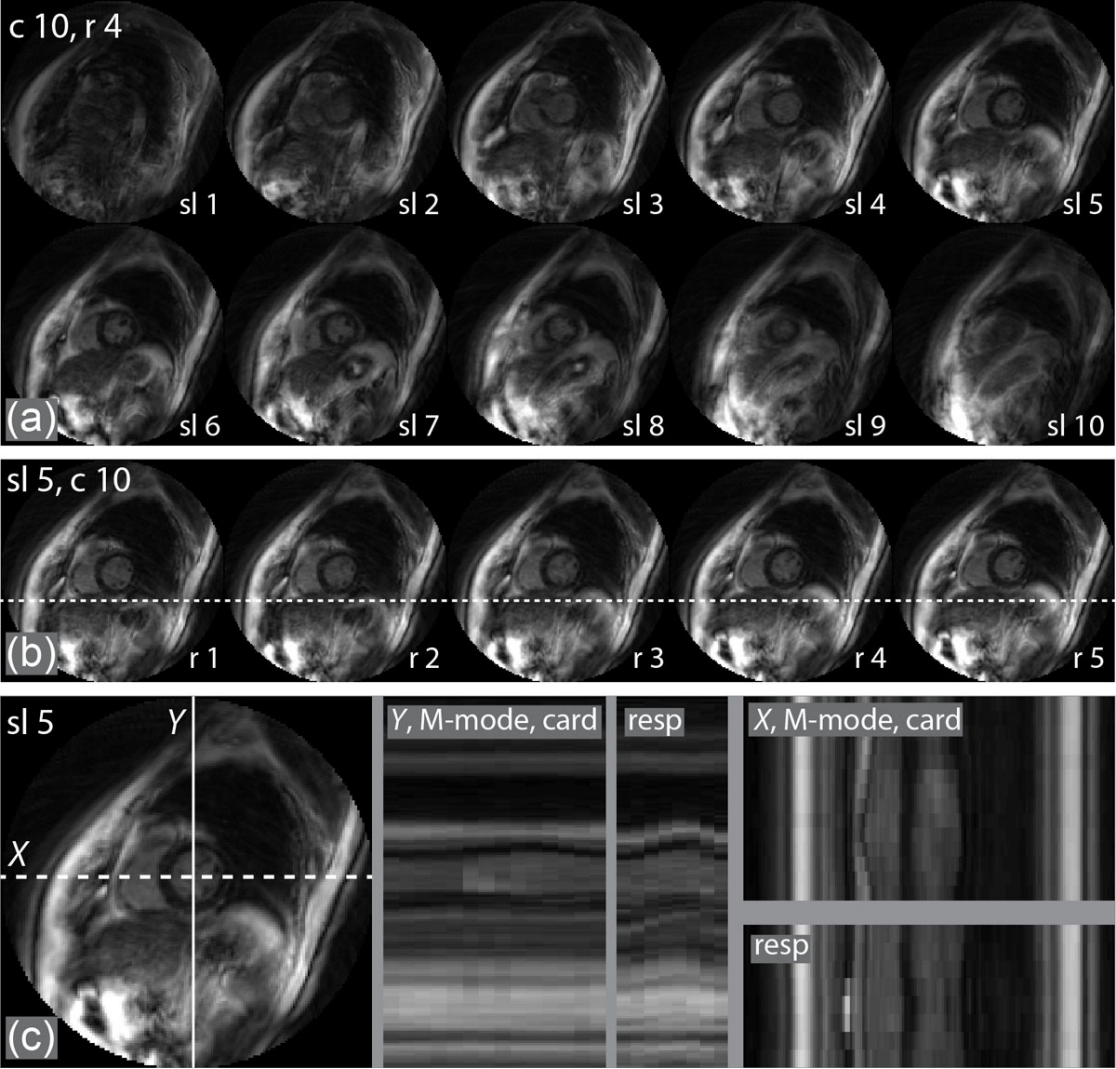
(a) All 10 slices from a healthy volunteer at cardiac phase 10/14 and respiratory phase 4/8. (b) First 5 frames from a *respiratory cine* of slice 5/10 at cardiac phase 10/14. (c) M-mode display of selected profiles *X* and *Y* in slice 5/10 with respect to cardiac or respiratory motion.

Fig. 2 shows representative data obtained from a healthy volunteer over 4 min 30 sec and reconstructed with 14 cardiac phases and 8 respiratory phases. A view of all 10 slices at cardiac phase 10 of 14 (end diastole) and respiratory phase 4 of 8 (end expiration) is displayed in Fig. 2a. In this view, we can re-display the 5D data as (1) a typical cardiac cine at a fixed respiratory phase, or (2) a *respiratory cine* at a fixed cardiac phase. An example of a *respiratory cine* of slice 5 and cardiac phase 10 is shown in Fig. 2b, where displacement of the diaphragm over respiratory phases 1 through 5 is readily appreciated. It is also useful to display a column of pixels in M-mode to visualize changes in the relative positions of chambers and interfaces over either the cardiac or respiratory cycle (Fig. 2c). Volumes and ejection fractions can also be measured with respect to respiratory-induced pressure changes and used to investigate diastolic dysfunction.

## Conclusions

Free-breathing 5D cardiac MRI is a rapid and robust technique for capturing a respiratory-resolved view of the heart. Respiratory cine and M-mode displays can help to visualize phenomena such as inspiratory septal shift in constrictive disease. Other display options, such as volume-rendered cines, are also possible.

## Funding

NIBIB T32 EB009035; NHLBI R01 HL039297; AHA 11POST7420014; GE Healthcare.

